# Maternal Impressions

**Published:** 1881-09

**Authors:** S. O. Stockslager

**Affiliations:** Boone, Iowa


### Domestic Correspondence.

ARTICLE VIII.

EDITORS MEDICAL JOURNAL AND EXAMINER: Seeing so many editorials on maternal impressions, brings to mind a case which strongly impressed me with the positiveness of maternal impressions.

I was called on May 23, 1881, to attend a multipara, she being healthy in every respect, but that she had burned her wrists some two months before, and that an old lady had told her that her baby would be marked just as she had been burned, that she had better have a doctor, and then, when the baby was born, the doctor could remove the marks, obliterating all traces of them. Therefore I was called. She was crying when I arrived—afraid her baby would be marked. I told her there was no likelihood of that, and to quiet herself, and not cross the bridge until she came to it. But, lo, when the child was born about two hours after, there was the exact counterpart of the burns on the mother. Although the mother's had entirely healed, the infant's looked like a burn done fifteen or twenty minutes afterward. I treated it just as a fresh burn, and it made a complete recovery without any marks remaining.

Does any one need any stronger evidence of the effect of maternal impression?

S. O. STOCKSLAGER, M.D.

BOONE, IOWA, July 21, 1881.

